# Remarkable antitumor effect of nivolumab in a patient with metastatic renal cell carcinoma previously treated with a peptide‐based vaccine

**DOI:** 10.1002/iju5.12139

**Published:** 2020-01-09

**Authors:** Ryoma Kurahashi, Takanobu Motoshima, Yumi Fukushima, Yoji Murakami, Junji Yatsuda, Takahiro Yamaguchi, Yutaka Sugiyama, Satoshi Fukushima, Yoshihiro Komohara, Shigetaka Suekane, Tomomi Kamba

**Affiliations:** ^1^ Department of Urology Faculty of Life Sciences Kumamoto University Kumamoto Japan; ^2^ Dermatology and Plastic Surgery Faculty of Life Sciences Kumamoto University Kumamoto Japan; ^3^ Cell Pathology Faculty of Life Sciences Kumamoto University Kumamoto Japan; ^4^ Department of Urology Kurume University School of Medicine Kurume Fukuoka Japan

**Keywords:** complete response, metastatic renal cell carcinoma, nivolumab, peptide vaccine

## Abstract

**Introduction:**

The safety and efficacy of combination therapy comprising immune checkpoint inhibitors and cancer‐specific peptide vaccines have not yet been established.

**Case presentation:**

A 71‐year‐old female metastatic renal cell carcinoma patient with multiple lung and pleural metastases. She had been treated with interferon alpha, sunitinib, axitinib, and pazopanib sequentially, but no clinical efficacy was observed. She participated in a clinical trial using cancer‐specific peptide vaccine therapy. Initially no antitumor effect was observed, and vaccine therapy was ceased after two courses. But 3 months after the start of nivolumab, remarkable tumor shrinkage was observed at all metastatic sites, which resulted in almost complete response at 6 months. At 10 months, nivolumab was stopped due to cellulitis at the peptide vaccine inoculation site. Intriguingly, even after nivolumab discontinuation, complete response was maintained for more than 1 year.

**Conclusion:**

We experienced a remarkable antitumor effect by nivolumab in a patient who was previously treated with vaccine therapy.

**Abbreviations & Acronyms**
CR: complete response
EGFR: epidermal growth factor receptor
HLA: human leukocyte antigen
IFNα: interferon alpha
irAE: immune‐related adverse event
LCK: lymphocyte‐specific protein tyrosine kinase
mRCC: metastatic renal cell carcinoma
MRP3: multidrug resistance‐associated protein 3
PD‐1: programmed cell death‐1
PD‐L1: programmed cell death‐ligand 1
SART2: squamous cell carcinoma antigen recognized by T cells 2
TIL: tumor infiltrating T cell

**Keynote message**

The safety and efficacy of combination therapy comprising immune checkpoint inhibitors and cancer‐specific peptide vaccines have not yet been established. However, the combined therapy could have a synergistic effect of compensating for each other’s weakness and exert a remarkable antitumor effect.

## Introduction

The treatment paradigm of mRCC has dramatically changed after the approval of anti‐PD‐1 monoclonal antibody nivolumab.^1^ However, there are some limitations, such as therapeutic effect is achieved in only 30% of mRCC patients and among 21% of patients experience grade 3–4 irAEs.^2^ Although peptide‐based vaccines enhance immunospecificity and immunogenicity against cancer, the immunosuppressive effect can be a problem.

Here, we report a case that started nivolumab as a later‐line treatment for mRCC following TKIs and peptide vaccine therapy, demonstrating remarkable tumor shrinkage that resulted in CR with a minor localized irAE.

## Case presentation

A 71‐year‐old woman underwent left radical nephrectomy 4 years ago. Pathological findings showed clear cell renal cell carcinoma G2 (pT3aN0M0), but 15 months after surgery multiple lung and pleural metastases occurred. Despite sequential therapy comprising IFNα, sunitinib, axitinib, and pazopanib, no clinical efficacy was observed and all drugs were discontinued due to cancer progression within 6 months.

During pazopanib treatment, the patient participated in a clinical trial to receive a cancer‐specific peptide vaccine at her own discretion. The patient's HLA subtype was HLA‐A24, and cancer‐specific peptides such as SART2, LCK, EGFR, and MRP3 were identified in renal cell carcinoma tissue (Table 1). These cancer peptides were used to eliminate cancer cells via activation and enhancement of the exhausted immune system to improve its potential to attack cancer cells. Administration of these cancer peptides was also expected to facilitate further elimination of remaining cancer cells by the immune system, resulting in a more favorable therapeutic effect. After two courses (18 times) of vaccine therapy, no significant anti‐cancer effect was detected. The patient subsequently decided to discontinue vaccine therapy, wished to receive newly approved nivolumab and was referred to our hospital.

**Table 1:** The immunoreactivity to SART2, LCK, EGFR, and MRP3 was activated by the peptide vaccine and it was maintained after vaccination. IgG response, as defined by FIU

The immunoreactivity of HLA‐A24 peptide subset					
HLA‐A24 peptides subset	Pre vaccination	Post 1st course	Post 2nd course	After 6 months	After 12 months
SART2‐93	62	5059	32 016	27 842	28 221
SART3‐109	30	28	19	14	12
Lok‐208	56	60	171	168	342
PAP‐213	32	28	23	15	13
EGF‐R‐800	62	163	10 738	5044	6335
MRP3‐503	18	419	13 704	7446	11 708
MRP3‐1293	44	52	52	33	37
SART2‐161	27	33	36	22	21
Lck‐486	55	24 602	24 724	18 549	20 116
Lck‐488	54	391	393	182	174
EZH2‐735	42	36	33	25	24
PTHrP‐102	40	80	38	25	21
				IgG(FIU)	

**Figure 1:**
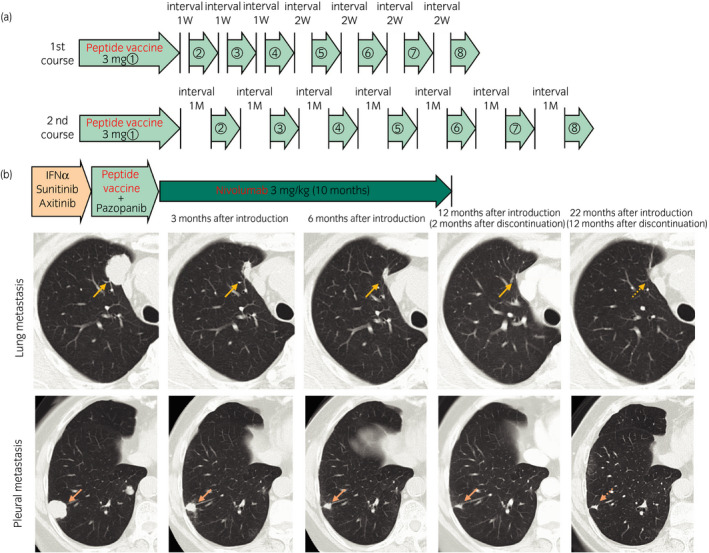
(a) Schedule of peptide vaccine administration. First course (8 doses/course): 3.0 mg/1.5 ml peptide vaccine is administrated by subcutaneous injection once per week four times, and the next four administrations are at the same dose once every 2 weeks. Second course: same dose is given once per month. (b) Chest computed tomography shows remarkable tumor shrinkage at lung and pleural metastatic sites after induction of nivolumab.

After obtaining informed consent about the possibility of severe irAEs, we started nivolumab at a dose of 3 mg/kg. Soon after the start of nivolumab, the multiple lung and pleural metastatic lesions began to shrink markedly and we finally achieved almost CR without any specific serious adverse events (Fig. 1).

**Figure 2:**
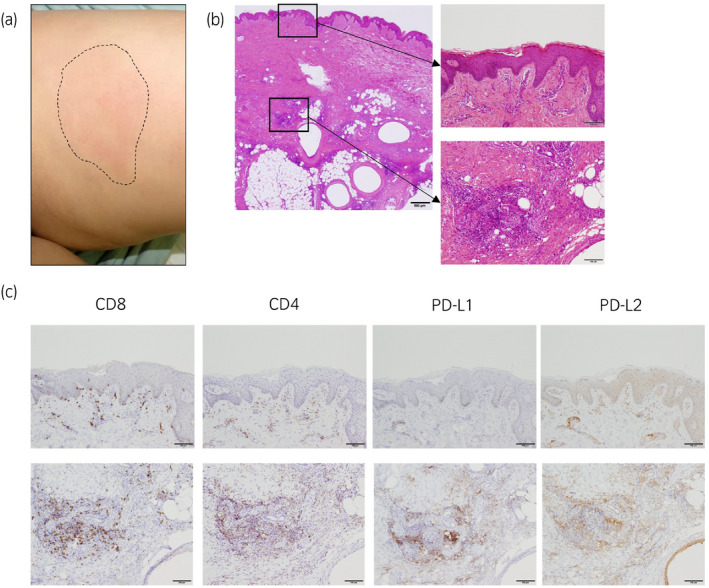
(a) Erythema and induration were observed on bilateral thigh corresponded to peptide vaccine inoculation sites. (b, c) Pathological findings of skin biopsy (H&E). Infiltration of lymphocytes, plasma cells, foam cells, and epithelioid cells indicates immune‐related cellulitis. Lymphocytes that infiltrated the erythema were CD8 dominant, and PD‐L1‐ and PD‐L2‐positive cells also infiltrated around the lymphocytes. Scale bar; 100 μm.

Ten months after the start of nivolumab administration, fever and erythema with induration over a 10‐cm area of bilateral thigh developed. Skin biopsy of the lesion was performed and pathological findings showed infiltration of a number of inflammatory cells, such as lymphocytes, plasma cells, foam cells, and epithelioid cells, resulting in a diagnosis of immune‐related cellulitis (Fig. 2). Interestingly, these skin reaction areas corresponded to peptide vaccine inoculation sites.

Because this irAE occurred, we ceased nivolumab treatment and continued close follow‐up without any anti‐cancer treatment. The patient has maintained CR without any clinical symptoms for more than a year since discontinuation of nivolumab.

## Discussion

Cancer vaccine therapy is one of the major immunotherapies, which induces specific anti‐cancer activity of lymphocytes through administration of specific cancer antigens.^3^ There are several methodologies, one of which is administering the identified cancer antigen peptide or protein with an adjuvant, and the other is transferring autologous lymphocytes that acquire tumor specificity by antigen stimulation.^4^, ^5^ In addition, another strategy is the use of an autologous cancer vaccine cell, which has been made more immunogenic by transferring a cytokine or chemokine gene.^6^ This treatment has had an excellent therapeutic effect for urological cancers, especially prostate cancer.^7^

Immune checkpoint inhibitors targeting molecules expressed in tumor cells and immune cells have strong modification effects. PD‐1 is expressed on activated T lymphocytes and functions through a checkpoint mechanism for T lymphocyte activation.^8^, ^9^ Immune checkpoint inhibitors, including nivolumab, release immune tolerance by acting directly on these target molecules, and introduce cytotoxicity to tumor cells.^10^

**Figure 3:**
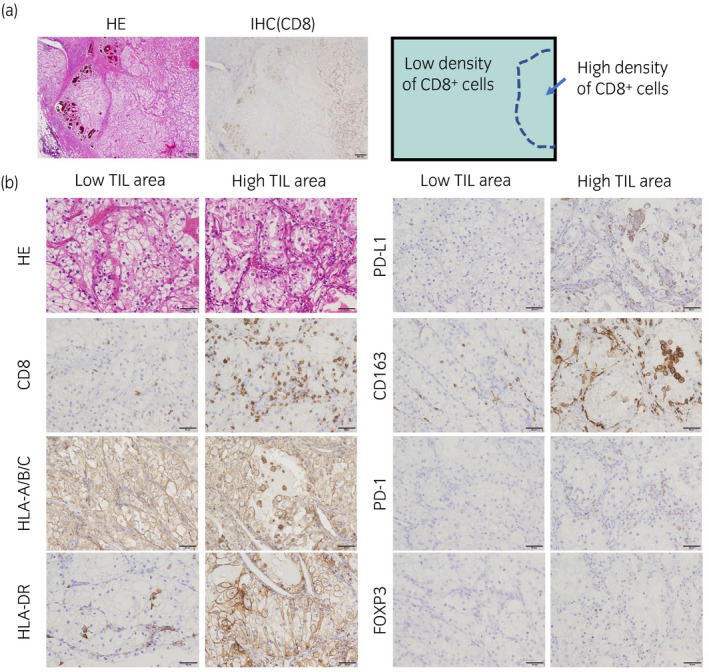
Histology of primary renal cell carcinoma. (a) Low magnification of hematoxylin and eosin (H.E.) staining and IHC of CD8 were presented. CD8‐positive TILs were focally detected. Scale bar; 500 μm. (b) H.E. and IHCs of low TIL area and high TIL area were presented. IHCs were performed using anti‐CD8 (clone C8/144B), anti‐HLA‐A/B/C (clone EMR8‐5), anti‐HLA‐DR (clone TAL1B5), anti‐PD‐L1 (clone 22C3), anti‐CD163 (clone 10D6), anti‐PD‐1 (clone EH33), and anti‐FOXP3 (clone 236A/E7) monoclonal antibodies. Scale bar; 50 μm.

In this case, there is a possibility that cancer‐specific T lymphocytes induced by cancer peptide vaccines were exhausted due to the expression of PD‐1. Such memory T‐cell exhaustion induced by the PD‐1 pathway was reactivated by nivolumab, resulting in strong and specific cytotoxicity with high‐affinity recognition of multiple cancer antigens induced by the peptide vaccine. This possibility is also supported by the skin reaction that occurred as an irAE was seen at vaccine inoculation sites. We confirmed that the infiltrating T lymphocytes are CD8‐dominant and PD‐L1‐ and PD‐L2‐positive cells infiltrating around the lymphocytes. It is highly likely that the remainder of the inoculated vaccine was later activated by nivolumab (Fig. 2). We also performed immunohistochemistry in the primary tissues. Cancer cells are all strongly positive for HLA‐class I (A/B/C), and focally positive for HLA‐DR. The density of CD8‐positive TIL was 3–10 cells/HPF in 90% area (low TIL area); however, high infiltration (more than 100 cells/HPF) of CD8‐positive TIL was seen in the HLA‐DR‐positive area. Increased numbers of PD‐1‐ and FOXP3‐positive cells were also detected in high TIL areas. PD‐L1‐positive cells and CD163‐positive M2‐like macrophages were also increased in high TIL areas (Fig. 3). Taken together, these results suggest that the induced CD8 lymphocytes may have been immunosuppressed by M2‐like macrophages such as TAM. Therefore, there were no tumor suppressive effects with peptide vaccine alone, and it seems that the antitumor effects were induced by administration of nivolumab.

Therefore, cancer vaccine therapy and immune checkpoint inhibitors can be expected to have a synergistic therapeutic effect compensating for each other's weakness with completely different mechanisms, exerting an antitumor effect. In fact, Ali *et al*.^11^ reported that combination therapy comprising an immune checkpoint inhibitor and cancer‐specific vaccine showed a remarkable tumor reduction effect in a melanoma xenograft murine model. In addition, randomized clinical trials are currently underway to examine the efficacy of this combination therapy in patients with melanoma and lung cancer (Clinical Trials.gov identifiers: NCT03047928 and NCT03406715).

When considering combination immunotherapy with immune checkpoint inhibitors and cancer‐specific vaccines, we have to pay attention to the possibility of severe irAE due to unexpected immune reactions such as excessive cytokine release leading to general inflammation.^12^ In this case, we started nivolumab after providing adequate information about such risks, and fortunately, there were no severe irAEs. Further study about the safety and efficacy of combined treatment comprising a peptide vaccine and immune checkpoint inhibitor is warranted.

## Conclusion

This is a unique clinical case that demonstrated a remarkable antitumor effect of nivolumab following previous treatment with a cancer‐specific peptide vaccine. Combination therapy comprising an immune checkpoint inhibitor with a cancer‐specific peptide vaccine could be a promising treatment option for patients with mRCC.

## Conflict of interest

The authors declare no conflict of interest.
